# Modulation of Cognition: The Role of *Gnidia glauca* on Spatial Learning and Memory Retention in High-Fat Diet-Induced Obese Rats

**DOI:** 10.1155/2019/2867058

**Published:** 2019-09-03

**Authors:** Wycliffe Makori Arika, Cromwell Mwiti Kibiti, Joan Murugi Njagi, Mathew Piero Ngugi

**Affiliations:** ^1^Department of Biochemistry, Microbiology and Biotechnology, School of Pure and Applied Sciences, Kenyatta University, P.O. Box 43844-00100, Nairobi, Kenya; ^2^Department of Pure and Applied Sciences, Technical University of Mombasa, P.O. Box 90420-80100, Mombasa, Kenya; ^3^Department of Environmental and Occupational Health, School of Environmental Sciences, Kenyatta University, P.O. Box 43844-00100, Nairobi, Kenya

## Abstract

Chronic exposures to high-fat diets are linked to neuropathological changes that culminate in obesity-related cognitive dysfunction and brain alteration. Learning, memory performance, and executive function are the main domains affected by an obesogenic diet. There are limited effective therapies for addressing cognitive deficits. Thus, it is important to identify additional and alternative therapies. In African traditional medicine, *Gnidia glauca* has putative efficacy in the management of obesity and associated complications. The use of *Gnidia glauca* is largely based on its long-term traditional use. Its therapeutic application has not been accompanied by sufficient scientific evaluation to validate its use. Therefore, the current study sought to explore the modulatory effects of dichloromethane leaf extracts of *Gnidia glauca* on cognitive function in the high-fat diet- (HFD-) induced obese rats. Obesity was induced by feeding the rats with prepared HFD and water *ad libitum* for 6 weeks. The *in vivo* antiobesity effects were determined by oral administration of *G. glauca* at dosage levels of 200, 250, and 300 mg/kg body weight in HFD-induced obese rats from the 6^th^ to the 12^th^ weeks. The Lee obesity index was used as a diagnostic criterion of obesity. The Morris water maze was employed to test spatial learning and memory retention in rats. The results indicated that *Gnidia glauca* showed potent antiobesity effects as indicated in the reduction of body weight and obesity index in extract-treated rats. Moreover, *Gnidia glauca* exhibited cognitive-enhancing effects in obese rats. The positive influences on cognitive functions might be attributed to the extracts' phytochemicals that have been suggested to confer protection against obesity-induced oxidative damage, reduction of central inflammation, and increased neurogenesis. The therapeutic effects observed suggest that *Gnidia glauca* might be an alternative to current medications for the symptomatic complications of obesity, such as learning and memory loss. Further studies are therefore needed to establish its toxicity profiles.

## 1. Introduction

Obesity is a significant modern health concern with widespread implications for individual health and well-being, family, and society [1]. The negative systemic effects of obesity on cardiovascular disease, metabolic physiology, and neuropsychological sequelae have attracted increased attention [2]. In rats, chronic exposure to a high-fat diet results in an obesogenic state that is coupled with positive impairment of the energy balance equation [3]. In particular, a high-fat diet linked with neuropathological changes culminates in obesity-related cognitive dysfunction and brain alterations [4, 5]. The cognitive domains mostly affected by an obesogenic diet include learning, memory performance, and the executive function [6–10]. These cognitive behaviors are mainly subserved by the hippocampus and the prefrontal cortex of the brain [11, 12].

The hippocampus is part of the limbic system bilaterally located in the medial temporal lobes of the brain critical for learning and memory processes [13]. It is highly susceptible to any endo- or exogenous insults [8]. Alterations in hippocampal morphology and function have implications for diverse behaviors such as cognitive flexibility, eating behaviors, working memory, emotional regulation, episodic memory, stress reactivity, spatial learning, and memory retention [13, 14].

Numerous preclinical studies have shown that chronic consumption of a high-fat diet is associated with cognitive decline, poorer cognitive performance, and an increased risk of depression, dementia, and Alzheimer's disease [4, 15, 16]. For instance, rats fed a 25% high-fat diet for 3 months demonstrated cognitive deficits in locating a hidden escape platform in an open-field water maze test [17]. Reduced cognitive faculties in the form of short-term memory and executive functioning are the main consequences of obesity [18]. The potential mechanisms underlying obesity-induced cognitive impairment include oxidative stress, central inflammation, brain atrophy, and breakdown of the blood-brain barrier [19].

Despite the remarkable progress in the management of obesity-induced cognitive impairment by synthetic drugs, there has been a renewed interest in medicinal plants. Medicinal plants are readily available, affordable, and biologically compatible, unlike the chemically synthesized drugs which have been associated with adverse effects and numerous health hazards [20]. Hereby, the identification and evaluation of active principles from herbal prescriptions have become the prime focus in the validation of their folklore use and drug discovery programs. The presence of phytochemicals in herbal medicines has been shown to enhance cognition through the activation of neurotransmitters on neurons [21], inhibition of beta-amyloid plaque formation [22, 23], and protection from neurotoxicity of amyloid *β*-peptide [24]. These phytobiotics exhibit free radical scavenging activities and sequester the insoluble protein aggregates in the brain [25]. They also confer anti-inflammatory effects and enhance cerebral blood flow [20, 25].

In traditional African medicine, *Gnidia glauca* has been therapeutically applied against many diseases such as sore throat, abdominal pain, wounds, burns, and snake bites [26]. It has also demonstrated significantly superior efficacy in the management of obesity and associated symptomatic complications such as anxiety, panic attacks, and cognitive impairments. The rationale for its utilization has rested largely on its long-term clinical experience. However, the continued upsurge in its use has not been accompanied by scientific evidence to support a traditional practitioner's claims. Therefore, the determination of cognitive-enhancing effects of dichloromethane (DCM) leaf extract of *G. glauca* in high-fat diet-induced obese rats is of prime focus in the validation of its folklore. Besides, since herbal medicines are viewed by the pharmaceutical industry as a source of “qualified leads” in the synthesis of modern drugs, the findings of this study will form a basis for the recruitment of *Gnidia glauca* as a candidate for drug design against cognitive deficits.

## 2. Materials and Methods

### 2.1. Collection of Medicinal Plant

Fresh leaves of *Gnidia glauca* were collected from their natural habitat at Makunguru Village, Nthawa Location, Siakago Division, Mbeere North Subcounty, Embu County, Kenya. The botanical identity of the plant was authenticated by a taxonomist and a voucher specimen deposited at the National Museums of Kenya Herbarium for future reference. The specimen was assigned a voucher number of WAM-V1. Coordinates for the locations of collection points were taken using a hand-held GPS machine model type Garmin etrex-H and recorded as shown in Table 1. The study was undertaken in the animal handling and experimental laboratory at the Department of Biochemistry, Microbiology and Biotechnology of Kenyatta University.

### 2.2. Processing and Extraction of the Plant Material

Fresh leaves of *G. glauca* were dried on a shade at room temperature for 21 days. The dried leaves of *G. glauca* were ground into fine powder by use of an electric mill. The milled plant sample was kept at room temperature free from direct sunlight in a dry airtight plastic container before extraction. In one liter of dichloromethane (DCM), 500 grams of the powdered sample of *G. glauca* was added and soaked for forty-eight hours. The dissolved compounds were decanted and filtered using a muslin cloth into a dry clean conical flask. The filtrate was concentrated under reduced pressure by use of a rotary evaporator at 40°C to attain a semisolid residue [27]. The yield of the plant extract was determined and subsequently refrigerated at -20°C before analysis.

### 2.3. Preparation of Appropriate Doses for Bioassays

The appropriate bioassay doses of DCM leaf extract of *G. glauca* for 5 animals per group were prepared by dissolving 0.23 g in 2.5 ml of 1% DMSO (200 mg/kg body weight), 0.29 g in 2.5 ml of 1% DMSO (250 mg/kg body weight), and 0.35 g in 2.5 ml of 1% DMSO (300 mg/kg body weight). Similarly, the dose of the reference drug, Orlistat, was prepared by dissolving 0.035 g in 2.5 ml of 1% DMSO (30 mg/kg body weight). The 1% DMSO was prepared by mixing 99 ml of PBS with 1 ml of 10% DMSO solution. In the entire dosing period, each experimental animal received a daily single-dose oral administration of 0.5 ml of treatments at 0800 hr. The choice for the oral route of drug administration was based on the fact that it mimics the commonly prescribed route of administration of *G. glauca* by herbalists. After the pilot study, the optimal activity of the extract was indicated at the doses of 200, 250, and 300 mg/kg body weight. All the treatment solutions were stored at -20°C until being used for bioassay.

### 2.4. Experimental Animals

Thirty female white albino Wistar rats of about eight to ten weeks weighing 120 ± 10 g were ordered from Kenya Medical Research Institute, Nairobi, Kenya. The rats were gonadally intact. Before the initiation of the experiment, the animals were randomly housed in groups of five in standard polypropylene cages which were maintained under controlled room temperature (23 ± 2°C), bench level lighting of 360 lux, and humidity (55 ± 5%) with a 12 hr light and 12 hr dark cycle for one week for acclimatization. The lights were turned on at 0700 and off at 1900 hr. During this period, the rats were fed a standard laboratory diet, in the form of rodent pellets from Unga Feeds Limited, Nairobi, Kenya, and water *ad libitum*. The rats were monitored thrice every day for health status. When no adverse events were indicated, the animals were weighed again before the initiation of the experiment. All procedures were carried out per the Public Health Service (PHS) Policy on Humane Care and Use of Laboratory Animals.

### 2.5. Obesity Induction

Obesity was induced by feeding the experimental animals with a high-fat diet and water *ad libitum* for twelve weeks. The composition of a high-fat diet was as provided by Levin and Dunn-Meynell [28] as indicated in Table 2. All ingredients of a high-fat diet were thoroughly mixed and baked in the oven at 65°C overnight. The normal rat chow diet was referred to as the control diet and given to negative control rats.

During the entire experimental period, the body weight of each rat was assessed in grams after every seven days using a digital Mettler PJ 3000 weighing balance.

The obesity index was defined by the Lee index. The Lee index was calculated according to the formula described by Lee [29].

(1) $$\begin{array}{c} \text{Lee index }\left(\%\right)=\sqrt[{3}]{\frac{\text{Body weight }\left(\text{g}\right)}{\text{Nose to anus length }\left(\text{cm}\right)}}\times 1000. \end{array}$$

Rats with a Lee obesity index value (equivalent to BMI ≥ 30 in humans) of 310 and above were considered obese [29] and used in the study. Following exposure to HFD (except for the normal control group) for 6 weeks, all the rats in the HFD group, HFD+Orlistat, and HFD+*G. glauca* extract-treated groups attained the target diagnostic value of obesity, indicating the end to the obesity induction phase. The obesity induction phase lasted for the first 6 weeks of the study while the treatment phase took the preceding 6 weeks. The naso-anal length (NAL) (cm) of rats was measured by a nonextensible thread and readings taken using a ruler with an accuracy of 0.1 cm.

### 2.6. Experimental Design

Thirty female rats were randomly grouped into 6 different sets of 5 animals each. Group 1 is the control (normal chow). Group 2 is the HFD alone. Group 3 is HFD+Orlistat. Groups 4-6 are HFD+*G. glauca* extract at 3 doses. All rats received water *ad libitum* throughout the study period.

### 2.7. Morris Water Maze

Spatial learning and memory retention (cognitive function) were determined using the Morris water maze (MWM) experiment [30, 31] to ascertain the effect of a six-week oral administration of *G. glauca*.

### 2.8. The Water Maze Apparatus

**Figure 1:**
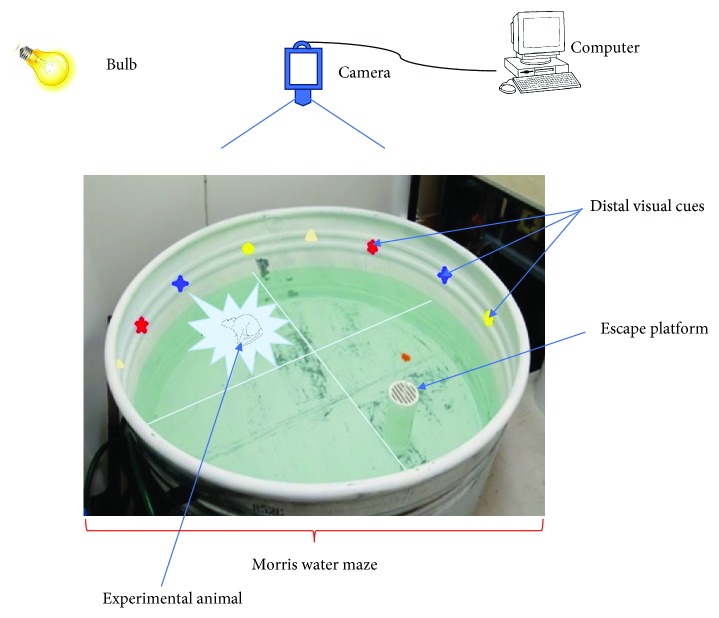
Morris water maze/navigation task.

**Figure 2:**
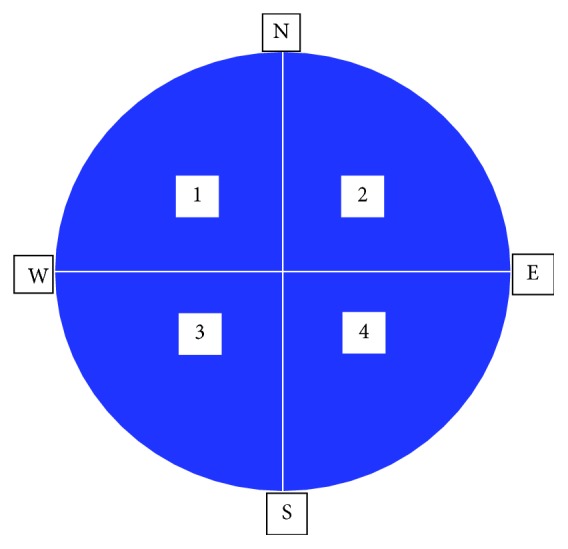
An illustration of the four quadrants of the water maze.

The water maze apparatus consists of a cylindrical metal barrel drum (pool) (diameter = 130 cm, height = 35 cm) (Figure 1). The pool was filled with water (22°C ± 2°C) and was made opaque by the addition of 1 kg of skim milk powder to ensure camouflage of the escape platform. A plexiglas cylinder with a stripped top (diameter = 9 cm, height = 20 cm) was used as the escape platform in the maze (Figure 1). The cylindrical escape platform was filled with water to weigh it down in the pool. The level of the water in the pool was adjusted to 1 cm below the surface of the striped top of the platform, thus creating a visible escape platform and to 1 cm above the striped top of the platform, thus creating an invisible escape platform. The pool was divided into four quadrants: northwest, northeast, southwest, and southeast, in the center of which a mark was made to ensure proper placement of the escape platform (branded 1-4) (Figure 2). Boundaries of these quadrants were marked on the edges of the pool with masking tape and labeled: north (N), south (S), east (E), and west (W) (Figure 2). Visible cues were mounted on the walls of the pool for orientation (Figure 1). Experimental sessions were captured by a video camera placed above the maze. The recorded trials were then fed to a detection system (HVS Image), which allowed tracking of the navigation paths and quantification of several parameters (Figure 1).

### 2.9. Procedure

The water maze test consisted of an acquisition phase, a reversal phase, and a probe trial phase that lasted ten days. A total of four trials were conducted for each replicate in each of the experimental group. The first day was an acquisition training with a visible platform followed by another acquisition training with an invisible submerged platform, for the 3 proceeding consecutive days. On day five, an acquisition probe trial was conducted with a no escape platform in the maze. On day six, reverse trials were conducted using the visible platform. Days 7–9 were reversal training days, again with an invisible platform. On the tenth day, a reverse probe trial was conducted with no escape platform.

### 2.10. Acquisition (Platform in Northwest Quadrant for Days 1, 2, 3, 4, and 5)

During acquisition training, the water level was adjusted appropriately such that the escape platform was submerged by 1 cm of water (invisible platform). The platform was positioned on the mark in the center of the northwest quadrant. Each animal received four trials of 60 seconds (max) per day. The starting positions of the animals were predetermined, which prevented any sequence of trials to be repeated by the same animal during any other day. The selected possible start positions were at the boundaries of the quadrants (such as west, north, east, or south). For each trial, the rats were placed in the water, facing the wall of the tank, in one of the four start locations. The rats were then permitted to explore the pool and to search for the hidden escape platform for 60 seconds. If the rats found the platform, the timer was stopped and the animal was permitted to remain on the platform for 15 sec. If the animal could not find the platform during the allotted time, the animal was guided onto the platform and allowed to remain on the platform for 15 sec to visually explore their surroundings. After each training session, the rats were dried with a towel and returned to their holding cage.

### 2.11. Reversal (Platform in Southeast Quadrant for Days 6, 7, 8, 9, and 10)

The invisible escape platform was moved to the opposite quadrant (Southeast quadrant), and rats were again assigned to appropriate start positions. The same procedures adapted in acquisition training were replicated during the entire period of reversal training.

### 2.12. Probe Trial (No Platform, Day 5 and Day 10)

To assess spatial memory retention ability, the animals were subjected to the 60 sec probe trial following the last training session of each phase (acquisition phase and reversal phase) upon the removal of the escape platform from the maze. The frequency of occupancy in the target quadrant was recorded.

### 2.13. Visible Platform (Visible Platform in Northwest Quadrant for Day 1 and Southeast Quadrant for Day 6)

To obtain a visible platform, the water level was adjusted appropriately such that the platform emerged 1 cm above the water surface. The visible platform was stationed in the northwest quadrant of the pool on day 1 in the acquisition phase while on day 6, the visible platform was in the southeast quadrant during the reversal phase. The same procedures as described above in both acquisition and reversal trainings were followed.

### 2.14. Running of Experimental Animals

During the entire experimental period, the animals were held in a cage lined with a paper towel to allow rats to dry. The paper towels were replaced when they become completely wet. Rats were then run sequentially per group with 5 minutes between each trial for each rat.

### 2.15. Gas Chromatography-Mass Spectrometry Analysis

Sample analysis was determined using GC-MS (7890/5975 Agilent Technologies Inc., Beijing, China) consisting of a gas chromatograph integrated into a mass spectrometer instrument. The GC-MS was equipped with a HP-5 MS (5% phenyl methyl siloxane) low bleed capillary column of 0.25 *μ*m film thickness, 0.25 mm diameter, and length of 30 m. An electron ionization system with ionization energy of 70 eV was used in GC-MS detection. A helium (99.99%) gas carrier was used at a consistent flow rate (1.25 ml/min) in a split mode. The mass transfer line and injector temperature were set at 200°C and 250°C, respectively. One microliter was used as an injection volume. Oven temperature was programmed from thirty-five degrees Celsius for five minutes, with an elevation of ten degrees Celsius per minute to two hundred and eighty degrees Celsius for 10.5 minutes, followed by fifty degrees Celsius per minute to two hundred and eighty-five degrees Celsius for 29.9 minutes with seventy minutes run time. The mass spectrometry operating conditions were as follows: ionization energy of 70 eV, ion source temperature of 230°C, relative detector gain mode scan speed of 1666 *μ*/sec, solvent cut time of 3.3 min, interface temperature of 250°C, and scan range of 40-550 m/z.

### 2.16. Data Management and Statistical Analysis

The data on time latency to reach the escape platform, swimming speed, navigation distance, and quadrant frequency was computed by the WatermazeBeta, Actimetrics Software, configured on an IBM PC-compatible computer. The data for each navigation variable was exported to Microsoft® Excel spreadsheet, where it was organized and later transferred to Minitab software version 17.1 for analysis. The data was found to conform to the assumptions of parametric data. One-way ANOVA was used to test the significance among the normal chow, HFD, HFD+Orlistat, and HFD+*G. glauca* extract-treated groups at a 99% confidence level. The data was further subjected to Tukey's post hoc for pairwise comparison and separation of means. The results were expressed as the mean ± standard deviation (SD) and presented in tables and graphs. Phytocompound identities were proposed based on their general fragmentation pattern using reference spectra published by the library-mass spectral databases [National Institute of Standards and Technology (NIST) library version (2005), software, Turbomas 5.2].

## 3. Results

### 3.1. Effects of Oral Administration of DCM Leaf Extract of *Gnidia glauca* on Body Weight of HFD-Induced Obese Rats

**Figure 3:**
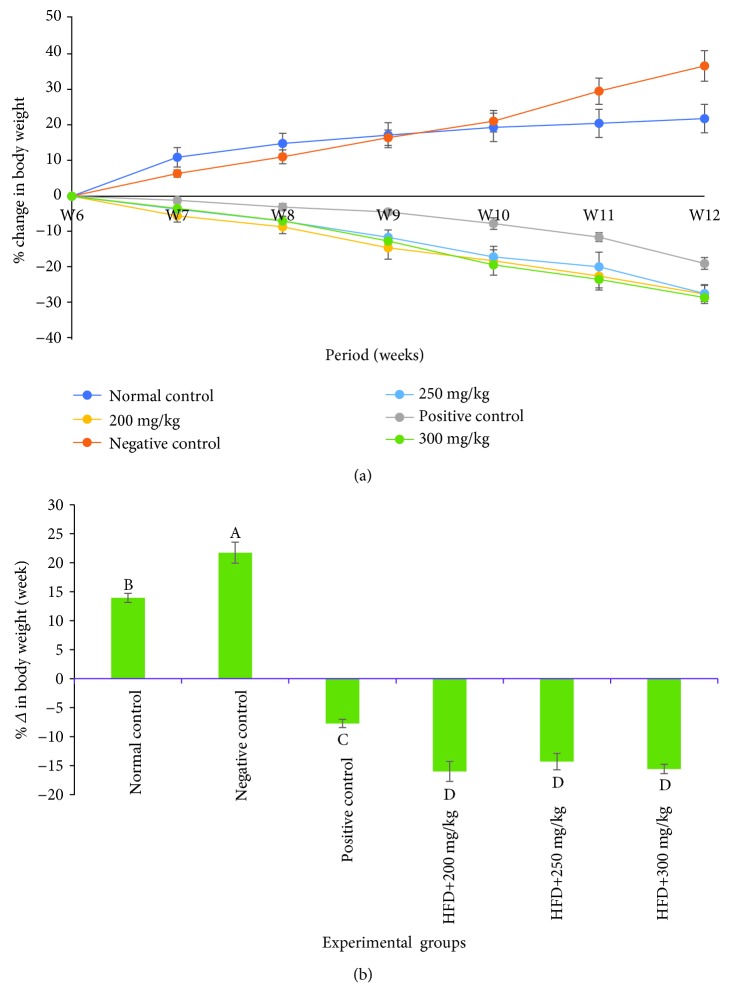
(a) Effects of DCM leaf extract of *Gnidia glauca* on body weights of HFD-induced obese laboratory rats per week. Each point on the curve represents the replicate measurement (*n* = 5) expressed as the mean ± SD of the data set at *p* ≤ 0.01. (b) Effects of DCM leaf extract of *Gnidia glauca* on the rate of change in body weights of rats per week. Each bar graph in the respective experimental group represents the replicate measurement (*n* = 5) expressed as the mean ± SD of the data set. The means with different letters across the experimental groups are statistically significant at *p* ≤ 0.01.

As depicted in Figure 3(a), the HFD group recorded a persistent increase in body weight throughout the study period. In contrast, the HFD+Orlistat and HFD+*G. glauca* extract-treated groups showed a reduction in body weight gain in the same period (Figure 3(a)). The analysis of the percentage change of the body weights of rats revealed that the HFD group and the normal chow group indicated a positive percentage change in body weight gain per week (Figure 3(b)). However, the increase in body weights of rats in the HFD group was higher than those of rats in the normal chow group. The HFD+Orlistat and HFD+*G. glauca* extract-treated groups showed a negative weekly percentage change in body weights throughout the study period (Figure 3(b)). Moreover, the HFD+*G. glauca* extract-treated groups indicated a higher rate of decrease in weight than the Orlistat-treated group of rats (Figure 3(b)).

### 3.2. Effects of Oral Administration of DCM Leaf Extract of *Gnidia glauca* on Obesity Index in HFD-Induced Obese Experimental Rats

**Figure 4:**
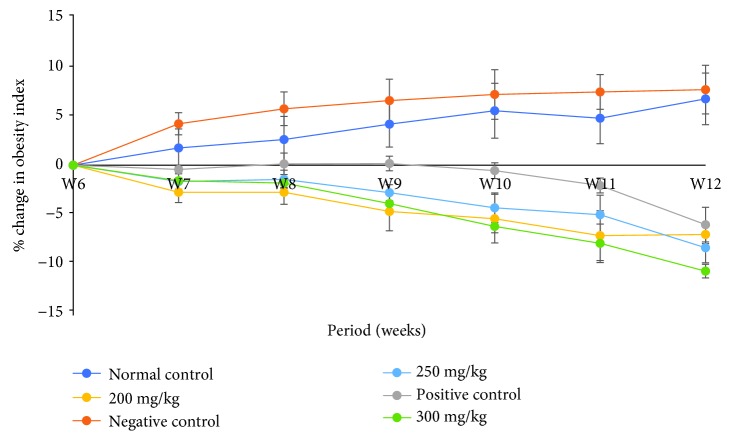
Effects of DCM leaf extract of *Gnidia glauca* on the obesity index of HFD-induced obese rats. Each point on the curve represents the replicate measurement (*n* = 5) expressed as the mean ± SD of the data set at *p* ≤ 0.01.

Results showed that chronic exposures of rats to high-fat diets caused an increase in the obesity index in the HFD group of rats throughout the study period (Figure 4). On the other hand, the treatment of rats with the reference drug, Orlistat, and the three doses of the plant extract caused a persistent decrease in the obesity index from the 6^th^ to the 12^th^ weeks of the study (Figure 4).

### 3.3. Analysis of the Navigation Behavior

The rats adopted the characteristic adult swimming posture of forepaws tucked under the head and hind legs used to propel it forward. The head largely remained above the water surface except for brief moments. Initially, the rats were thigmotaxic, swimming around the perimeter of the pool near or against the sidewalls, making occasional efforts to escape by forepaw climbing movements against the side. Later, they swam out into open water, crossing the pool several times during each of the initial habituation sessions. The rats readily climbed onto the escape platforms when they encountered them or were guided to locate the platform when they failed to locate it within the experimental period. Regularly, the rats would then rear and/or turn around for a few seconds before making vigorous “wet-dog” shakes followed by grooming and occasional face-washing. There was no sign of the animals treating the “underwater” platform any differently from the “above-water” platform as a refuge from the water. All rats exhibited similar navigation behavior independent of treatment or exposure to diet.

### 3.4. Latency Period

#### 3.4.1. Acquisition Training

On the first day of the visible platform tests, the HFD group exhibited a longer latency period to escape onto the visible platform relative to the HFD+Orlistat and HFD+*G. glauca* extract-treated groups (Table 3). In the hidden platform tests (2^nd^-4^th^ days), the HFD+Orlistat and HFD+*G. glauca* extract-treated groups showed a shorter mean escape latency period onto the hidden platform than the HFD group (Table 3). The HFD group showed considerably poorer performance throughout the acquisition period (Table 3).

#### 3.4.2. Reverse Training

When the escape platform was moved to the opposite quadrant, the HFD+Orlistat and HFD+*G. glauca* extract-treated groups showed a reduction in the latency period onto the escape platform (in both the visible and invisible platform tests) relative to the HFD group (Table 4). Further, it was observed that HFD+Orlistat and HFD+*G. glauca* extract-treated groups showed a persistent decrease in search time throughout the testing sessions (Table 4).

### 3.5. Navigation Distance

#### 3.5.1. Acquisition Training

The analysis of the navigation distance exhibited that the HFD+Orlistat, normal chow, and HFD+*G. glauca* extract-treated groups covered shorter distances to reach both the visible and invisible escape platforms than the HFD group (Table 5). It was also noted that there was no difference in swimming path lengths among the normal chow, HFD+Orlistat, and HFD+*G. glauca* extract-treated groups during the acquisition training (*p* > 0.01; Table 5).

#### 3.5.2. Reverse Training

The reverse trials yielded a considerable trial-to-trial variability among the groups with the HFD group covering a longer distance to reach both the visible and invisible escape platforms relative to the HFD+Orlistat, normal chow, and HFD+*G. glauca* extract-treated groups (Table 6), learning about the new location of the platform then proceeded very rapidly in the HFD+Orlistat and HFD+*G. glauca* extract-treated groups as indicated in the decrease in their navigation distances (Table 6).

### 3.6. Swimming Speed

#### 3.6.1. Acquisition Training

The analysis of the speed variable indicated that the HFD+Orlistat and HFD+*G. glauca* extract-treated groups swam relatively faster than the HFD group to reach the escape platforms in both visible and invisible trials (Table 7). It was also noted that the swimming speed of HFD+*G. glauca* extract-treated groups was independent of the extract's concentrations (Table 7).

#### 3.6.2. Reverse Training

The set of analyses that were performed on these sessions yielded a similar pattern of results as those of the acquisition test (Table 8). The HFD+Orlistat and HFD+*G. glauca* extract-treated groups swam faster than the HFD group (Table 8). The analysis also yielded a session effect which indicated that swimming speed increased over the course of the trials thereby decreasing the swim latency period in the HFD+Orlistat and HFD+*G. glauca* extract-treated groups (Table 8).

### 3.7. Spatial Memory Retention

#### 3.7.1. Acquisition

**Figure 5:**
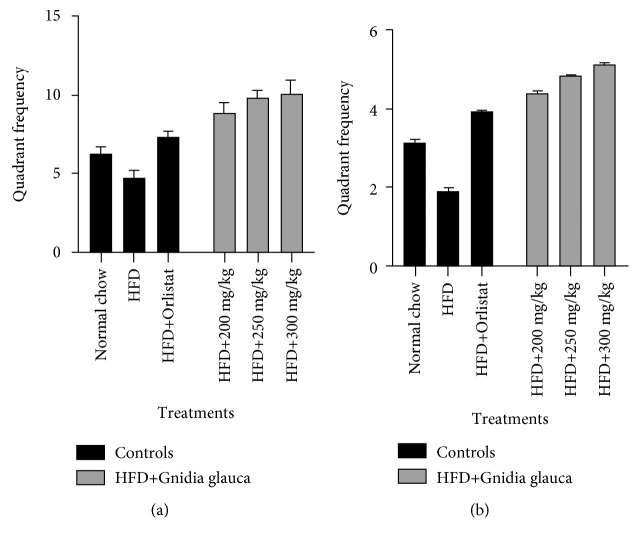
(a) Effect of DCM leaf extract of *G. glauca* on spatial memory retention in HFD-induced obese rats during acquisition training. The criterion for significance was set at *p* ≤ 0.01. (b) Effect of DCM leaf extract of *G. glauca* on spatial memory retention in HFD-induced obese rats during reverse training. The criterion for significance was set at *p* ≤ 0.01.

The analysis performed on the probe test on the 5^th^ day in which the platform was withdrawn from the water indicated that the HFD+Orlistat and HFD+*G. glauca* extract-treated groups exhibited preference for the northwest quadrant (which was the correct location of the escape platform during the acquisition training) than the HFD group (Figure 5(a)). Besides, the HFD+*G. glauca* extract-treated group exhibited high frequency for the correct quadrant in a dose-dependent manner. The quadrant frequency in the HFD+*G. glauca* extract-treated group was higher than that observed in both the HFD+Orlistat and normal chow groups (Figure 5(a)). The HFD+Orlistat group showed no much difference in spatial bias towards the target quadrant as was observed in the normal chow (*p* ≤ 0.01; Figure 5(a)).

#### 3.7.2. Reverse Training

During the reverse trial (on the 10^th^ day), it was observed that the HFD+Orlistat and HFD+*G. glauca* extract-treated groups showed a higher number of entries into the correct quadrant (southeast quadrant) relative to the HFD group (Figure 5(b)). The increase in the number of entries into the correct quadrant was in a concentration-related manner in the HFD+*G. glauca* extract-treated group. Besides, results showed that rats in the HFD+*G. glauca* extract-treated group concentrated their search in the quadrant where the platform was previously located during the training sessions more than rats in the normal chow group (Figure 5(b)).

#### 3.7.3. Relative Abundance of Bioactive Compounds in DCM Leaf Extract of *G. glauca*

The gas chromatography-mass spectrometry analysis (GC-MS) of the DCM leaf extract of *G. glauca* indicated the presence of 17 compounds (Table 9). Based on the analysis, oleic acid (21.05 ± 2.34%) was the most abundant followed by *γ*-sitosterol (18.84 ± 1.04%), curcumin (16.91 ± 2.30%), quercetin (15.74 ± 1.01%), stilbenes (13.39 ± 4.06%), vitamin E (12.25 ± 1.67%), octadecanoic acid (stearic acid) (10.73 ± 1.55%), and others (Table 9).

## 4. Discussion

The Morris water maze is a versatile behavioral tool used to test cognitive processes such as spatial learning and memory retention in laboratory rodent models [14]. The experimental animals rely on extramaze cues to navigate around an open swimming arena from identified start points to locate a submerged invisible escape platform [32]. To assess spatial learning, repeated trials are practiced while reference memory is determined by a probe test when the escape platform is absent in the target quadrant [33].

High-fat diet-induced obesity increases the risk of development of cognitive impairment in the form of short-term memory, executive function deficits, dementia, and Alzheimer's disease [7, 34]. Short-term exposure to an obesogenic diet even for only 72 hours is sufficient to impair hippocampal-dependent memory [35, 36]. The hippocampal-dependent spatial learning examines the ability of an experimental animal to acquire spatial information by measuring various variables such as escape latency, swimming speed, and navigation distance [32]. The spatial memory retention examines the ability of an animal to recall the correct location of the escape platform in the maze [32].

The current study is aimed at evaluating the effect of *G. glauca* on memory and learning processes in HFD-induced obese rats. The results showed that chronic exposures to high-fat diets resulted in obesogenic states as evidenced by the increased body weight and Lee obesity index. The increase in body weight could be due to a high rate of acylation of saturated fatty acids into triglycerides that are subsequently stored in the adipose tissues [37]. The obesity index has been established to be the best predictor of intra-abdominal fat in rats and, therefore, of central obesity [38]. The Lee index (∛weight/naso − anal length × 1000) has been shown to correlate with fat mass. Even though the naso-anal length is a relatively weak predictor of fat-free mass in rats, the Lee index is currently used as a fast and accurate way to access obesity in rodents subjected to a weight gain method [39].

The results also showed that chronic exposures to high-fat diets directly influences the spatial learning and memory function of rats in the maze. It was observed that the HFD group indicated longer latency to escape onto the hidden platform, swam slower, and covered larger distances to reach the submerged platform in both acquisition training and reversal trials. Also, rats in the HFD group searched the escape platform rather randomly and spent more time in nontarget quadrants in the maze, an indication of their inferior memory of the learning task. Similar studies have demonstrated that rodents fed high-fat diets portray marked impairments in cognitive functions as determined by latency to find the hidden escape platform [40], their swimming speed, navigation distance, and probe trials in the Morris water maze [41, 42].

The cognitive variables such as escape latency and navigation distance reflect the integrity of the hippocampus to locate the hidden escape platform and ought to translate in shorter time latency and path distances [43]. The navigation speed is influenced by the weight of an animal and, therefore, reflects the perceptive or motor capacities of the experimental animals [43]. Spatial learning and memory retention as determined by the probe test demonstrate whether navigation behavior towards the escape platform depends on allocentric cues [43].

Chronic exposure to HFD is often associated with hyperglycemia due to insulin insensitivity [44]. One of the metabolic complications associated with persistent high blood sugar levels is retinopathy, which renders poor vision [45]. The dismal performance of rats in the HFD group could be attributed to poor vision, which renders them to inappropriately perceive the surrounding external cues that are necessary to locate the escape platform. These observations corroborate with findings of Bélanger et al. [43], whose 8-week untreated diabetic ZDF rats developed cataracts, which consequently resulted in poor performance in the maze.

The observation that the HFD group swam slower indicates impaired motor functions. The altered motor capacities of these obese rats could be due to their overweight condition. A study by Frisbee and Stepp [46] showed that the muscular tissues of ZDF rats deteriorate faster in comparison to those of normal control rats. Several studies on *Rosmarinus officinalis* (Rosemary) [47], *Bacopa monnieri* [48], and *Centella asiatica* [49] have also demonstrated an increase in ambulatory activities, enhancement of cognition, and positive modulation of mood, anxiety, and depression disorders.

Chronic exposure to calorically dense foods has been implicated to contribute to cognitive impairment, dementia, and Alzheimer's disease [50]. The potential mediators underlying obesity-induced cognitive decline include brain atrophy, breakdown of blood brain barrier (BBB), systemic and central inflammation, and oxidative stress [50]. Increased adiposity has shown a positive correlation with reduced hippocampal volume and cognitive decline [51]. A high dietary fat was shown to induce hippocampal-hypothalamic neuronal apoptosis resulting in the reduction in hippocampal weight [52, 53]. Besides, high-fat diets decrease the levels of the hippocampal brain-derived neurotrophic factor (BDNF), which mediates neuronal changes involved in learning, memory, neurogenesis, and synaptic plasticity [54]. The metabolic and dietary consequences of a high-fat diet intake influence the brain function by disrupting the integrity of the BBB [55]. The obesity-induced BBB dysfunction, neuronal impairment, and memory loss are the frequent consequences of plasma accumulation of amyloid proteins (A*β*) that pathologically affects the cerebrovasculature [56].

Systemic and central inflammation have been shown to contribute to cognitive decline via cytokine-mediated production [57]. The synthesis and release of proinflammatory cytokines such as IL-1*β*, IL-6, and TNF-*α* contribute to cognitive impairment by affecting cognitive processes such as synaptic plasticity, neurogenesis, neuromodulation, memory consolidation, and long-term potentiation (LTP) [58]. Chronic consumption of a high-fat diet exacerbates oxidative damage in the hippocampus due to attenuated antioxidant defenses and facilitates the production of proinflammatory cytokines, chemokines and brain's resident immune cells, the microglia, and astrocytes [59, 60]. These events, therefore, culminate in impaired neurogenesis, synaptic remodeling, and reduction in neuronal spine density as well as neuronal apoptosis, deregulated HPA axis, neurodegeneration, and brain atrophy [61, 62].

Treatment of rats with the standard drug, Orlistat, and the three doses of *G. glauca* caused a decrease in body weight and Lee obesity index. Orlistat is a saturated derivative of lipstatin that inhibits the activity of pancreatic lipase resulting in reduced dietary fat absorption [63]. The antiobesity effects of the extract might also be attributed to the reduction of triglyceride absorption through the inhibition of the action of pancreatic lipase. The extract might have also led to an increase in energy expenditure, inhibited the differentiation and proliferation of preadipocytes, and stimulated satiety signals (such as leptin) thereby resulting in the reduction of body and obesity index [64].

The results also showed that the HFD+Orlistat and HFD+*G. glauca* extract-treated groups recorded shorter latency to escape onto the hidden platform, swam faster, and covered shorter distances to reach the submerged platform in the maze. In the probe test, the same group of rats showed a novel behavioral strategy of concentrating their search in the target quadrant, where the platform was located in the previous training sessions. These behaviors suggest that the treatment of rats with the standard drug, Orlistat, and the three extract doses improves obesity-induced memory impairments. Consistent with this study, it was reported that the high-fat and high-fructose diet-induced obese mice treated with *Camellia sinensis* (green tea) showed lower latency and escape distance than the high-fat and high-fructose diet-induced obese untreated mice on each test day. Besides, the treated mice spent a long time in the target quadrant and had greater numbers of platform crossings than the untreated obese mice [65]. Since navigation towards the escape platform is based on allocentric cues, Orlistat and the plant extract might play a role in the improvement of vision through amelioration of metabolic complications of obesity-induced type 2 diabetes.

The probable mechanisms attributed to the positive influences on cognitive functions by *G. glauca* among others include the normalization of antioxidant mechanisms [66], reduction of inflammation [67, 68], increment in the expression of hippocampal neurotrophic factors (BDNF), and enhancement of neurogenesis which facilitates neural plasticity in the hippocampus [69, 70].

The cognitive-enhancing ability of *G. glauca* may be due to the presence of phytochemicals which have been implicated in the improvement of learning and memory [38]. The GC-MS analysis revealed the presence of various bioactive compounds such as polyphenols (luteolin, catechins, curcumin, quercetin, anthocyanins, and naringenin), alkaloids, long-chain polyunsaturated fatty acids, and Vitamin E. The normal chow pellet also contains appreciable amounts of these phytochemicals such as catechins, flavanols, quercetin, and vitamins (B, D, and E). These phytocompounds confer multiple physiological effects that not only serve to protect the brain from the pathogenic but also reverse the underlying disease process [38]. The normalization of cognitive effects in extract-treated groups as that observed in the normal chow group could be attributed to the synergistic and/or additive effects of these biocompounds.

Luteolin has been shown to ameliorate obesity-induced cognitive impairments [71]. The administration of luteolin to high-fat diet-induced obese mice decreased the circulating levels of serum adipocytokines, alleviated neuroinflammation, and reduced neuronal insulin resistance [71]. Luteolin increased the levels of brain-derived neurotrophic factor (BDNF) and improved oxidative stress-mediated cognitive decline [71]. Luteolin was also shown to enhance the action of synapsin I (SYP-1) and postsynaptic density protein 95 (PSDP-95) in the hippocampus and medulla cortex of the brain [71].

Catechins such as epigallocatechin-3-gallate have been implicated to have the potential to alleviate high-fat and high-fructose-induced insulin resistance [71]. It has been shown to improve cognitive impairment in mice [71]. The treatment of high-fat and high-fructose-induced mice with catechins significantly increased the average time spent in the target quadrant [65]. The treated mice also recorded a greater number, in platform crossings, than their obese-untreated counterparts, an indication of improved memory [65, 71]. Catechins exhibit their protective effects by preventing A*β*-induced neuronal injury through scavenging of ROS [72]. Specifically, catechins decrease the levels of malonyldialdehyde (MDA) and caspase, thereby resulting in decreased ROS [72]. Catechins inhibit fibrillogenesis of A*β* through their direct binding to the unfolded polypeptides A*β*, thereby converting them to unstructured, nontoxic A*β*-oligomers instead of *β*-sheet-rich aggregates [73].

Stilbene such as resveratrol is a polyphenolic compound which has been reported to reduce the accumulation of amyloid plaques (A*β* peptide) in Tg2576 neuron cultures [74]. Amyloid plaques trigger microglial activation by interacting with toll-like receptors (TLR) such as TLR4. Activated microglia induce neuronal inflammation and cell death [74]. Therefore, the anti-inflammatory activities of stilbenes protect microglia against A*β*-induced inflammation [75].

Quercetin is a flavonoid exhibiting antioxidant, antiapoptotic, and anti-inflammatory properties [76]. Quercetin was reported to confer cognitive-enhancing effects through reduction of *β*-amyloid plaque aggregation [76]. Quercetin treatment of 3xTg Alzheimer's disease mice decreased IL-1*β*/COX-2/iNOS proinflammatory signaling in the hippocampal CA1 region [77].

Curcumin is a natural polyphenol whose supplementation in the diet has been reported to ameliorate HFD-induced cognitive deficits [78]. Curcumin modulates cognition by improving synaptic plasticity through alterations of the N-methyl-D-aspartate receptor (NMDAR) and calcium/calmodulin-dependent kinase II (CaMKII) [79]. Curcumin reduces oxidative stress and promotes the synthesis of docosahexaenoic acid (DHA) from its precursor, *α*-linolenic acid, by stimulating the activity of enzymes involved in the synthesis of DHA such as elongase and fatty acid desaturase-2 (FADS-2) in the brain [80, 81].

Anthocyanins (ANTs) have been implicated in the reduction of A*β*-induced neurotoxicity by reducing ROS formation upon exposure of A*β*1-40 and A*β*25-35 to neuro-2A cells [82]. Anthocyanins also reduce A*β*-induced neurotoxicity through perturbation of calcium balance and inhibition of metabolism of apolipoprotein E (ApoE) [82]. Anthocyanins promote the formation of nontoxic forms of A*β* aggregates instead of the toxic amyloid fibrils by direct binding to A*β* molecules, thereby suppressing amyloid fibril formation [83].

Naringenin chalcone neuroprotective effects are well characterized. It enhances learning and memory ability in mice by reducing senile plaque formation and increasing glucose uptake in the brain [84]. Naringenin enhances cognition through inhibition of GSK3*β* activity and mitigation of mitochondrial dysfunction mediated oxidative stress [85]. It also enhances cognition by stimulating the activity of CaMKII and suppression of acetylcholinesterase activity [85, 86].

Alkaloids isolated from *Huperzia serrata* were shown to be a potent, reversible, and selective inhibitor of acetylcholinesterase (AChE) [87]. Alkaloids exhibit memory-enhancing efficacy due to their ability to penetrate the blood-brain barrier and inhibit acetylcholinesterase action [87].

Previous studies have reported that vitamin E is an important component of the body antioxidant systems. Its antioxidant activity and anti-inflammatory properties contribute to its neuroprotective effects [88]. Vitamin E inhibits A*β* accumulation in the brain. Moreover, the reduction of oxidative stress by vitamin E protects against phosphorylation of A*β*-induced tau through the inhibition of the activation of p38-MAPK [89].

Long-chain polyunsaturated fatty acids such as omega-3 fatty acids (such as alpha-linolenic acid, docosahexaenoic acid, and eicosapentaenoic acid) and omega-9 fatty acid (oleic acid) have been implicated in the improvement of cognitive deficits [90]. Omega-3 and omega-9 fatty acids are predominantly found in the brain [90]. A low dietary ratio of omega-3/omega-6 is linked with cognitive impairments and dementia [91, 92]. The dietary omega-3/omega-6 ratio is inversely correlated with cognitive decline and hippocampal inflammation [93]. Omega-3 supplementation increases molecular markers involved in neuronal plasticity such as BDNF and tropomyosin receptor kinase B (TrkB) [94]. Mice feeding with a high-omega-3/omega-6 ratio diet showed low mRNA expression levels of hippocampal inflammatory markers such as TNF-*α* and IL-1*β* [95]. Therefore, PUFA contributes to the prevention of neuroinflammatory processes [93].

## 5. Conclusion

The present study focused on the determination of cognitive-enhancing effects of the DCM leaf extract of *G. glauca* in HFD-induced obese rats. Results showed that the DCM leaf extract of *G. glauca* ameliorates learning and memory deficits in HFD-induced obese rats. The hippocampal-dependent spatial learning examines the ability of the experimental animal to acquire spatial information by measuring various variables including escape latency, swim speed, and navigation distance. Spatial memory retention is assessed using the probe test in the Water maze. The improvement of cognitive variables (escape latency, swim speed, and navigation distance as well as probe trial) in the DCM leaf extract of *G. glauca*-treated rats relative to the HFD group obese rats are indicative of extracts' potential to treat cognitive deficits. The positive influences on cognitive functions could relate to the antiobesity effects of the extract. The cognitive-enhancing ability of the extract might be attributed to its capacity to confer protection against obesity-induced oxidative damage, restoration of redox homeostatic status, and reduction of central inflammation. Also, the extract might have enhanced the gene expression of hippocampal neurotrophic factor (BDNF) and increased neurogenesis, thereby facilitating neuronal plasticity in the hippocampus. The therapeutic effects of the extract are mainly attributed to the phytochemicals present. The learning and cognitive-enhancing effects of *G. glauca* leaf extract might also have a positive implication in the management of dementia and Alzheimer's disease. This study, therefore, provides a basis for further research on *G. glauca* as a plant-derived source of a drug against symptomatic complications of obesity such as learning and memory loss. However, there is a need to conduct comprehensive toxicity studies to establish the safety profile of this plant.

## Figures and Tables

**Table 1:** Coordinates of the site of collection of the plant sample.

Plant species	UTM eastings	UTM northings	Latitude DMS	Longitude DMS
*G. glauca*	348,712.48	9,936,131.99	0^o^34′39.61^″^S	37^o^38′25.72^″^E

The coordinates of the location where the *Gnidia glauca* was collected.

**Table 2:** Composition of high-fat diet and normal rat chow diet.

High fat diet	
Nutrients	%/100 g
Carbohydrate	43
Protein	17
Fat	40
Ingredients	g/100 g
Powdered rat feed	68.0
Maize oil	6.0
Ghee	6.0
Milk powder	20.0
Total energy (kcal/100 g)	514.0

Normal rat chow diet	
Nutrients	%/100 g
Carbohydrate	48.8
Protein	21
Fat	3
Calcium	0.8
Phosphorus	0.4
Fiber	5
Powdered rat feed	68
Ash	8
Total energy (kcal/100 g)	356.2

Nutrients and ingredients of high-fat diet and normal rat chow diet.

**Table 3:** Effect of DCM leaf extract of *Gnidia glauca* on escape latency in HFD-induced obese rats during acquisition training.

Treatment (mg/kgbw)	Escape latency (sec)			
	Day 1	Day 2	Day 3	Day 4
Normal chow	35.20 ± 0.84^b^	43.60 ± 2.07^b^	32.20 ± 1.30^b^	24.20 ± 1.30^b^
High-fat diet	46.80 ± 1.30^a^	53.20 ± 0.84^a^	45.80 ± 1.48^a^	36.00 ± 1.58^a^
HFD+Orlistat	32.80 ± 1.92^b^	43.40 ± 2.79^b^	32.60 ± 2.07^b^	22.40 ± 0.89^b^
HFD+200 mg/kg	33.20 ± 2.17^b^	41.00 ± 2.00^b^	30.80 ± 1.64^b^	20.00 ± 1.00^c^
HFD+250 mg/kg	33.40 ± 1.52^b^	40.60 ± 1.14^b^	29.60 ± 1.52^b^	17.80 ± 1.30^cd^
HFD+300 mg/kg	33.80 ± 0.84^b^	40.20 ± 0.84^b^	30.00 ± 1.87^b^	16.00 ± 0.71^d^

Results are expressed as the means ± SD for five rats per group. The means within the respective columns followed by similar superscript are not significantly different at *p* ≤ 0.01, analyzed by ANOVA followed by Tukey's post hoc test for multiple comparison.

**Table 4:** Effect of DCM leaf extract of *Gnidia glauca* on escape latency in HFD-induced obese rats during reverse training.

Treatment (mg/kgbw)	Escape latency (sec)			
	Day 6	Day 7	Day 8	Day 9
Normal chow	40.80 ± 0.84^b^	48.60 ± 0.55^b^	38.80 ± 1.30^b^	28.80 ± 0.84^b^
High-fat diet	50.60 ± 0.55^a^	57.60 ± 1.14^a^	46.80 ± 1.92^a^	38.60 ± 1.14^a^
HFD+Orlistat	37.40 ± 1.14^c^	46.20 ± 1.92^bc^	35.60 ± 1.14^c^	26.60 ± 1.14^bc^
HFD+200 mg/kg	37.40 ± 1.52^c^	45.80 ± 2.59^bc^	35.80 ± 0.84^c^	24.60 ± 1.34^c^
HFD+250 mg/kg	38.20 ± 0.84^c^	44.80 ± 0.84^c^	34.60 ± 1.52^cd^	21.40 ± 1.34^d^
HFD+300 mg/kg	38.60 ± 1.14^c^	45.00 ± 1.00^c^	32.00 ± 1.58^d^	19.60 ± 0.89^d^

Results are expressed as the means ± SD for five rats per group. The means within the respective columns followed by similar superscript are not significantly different at *p* ≤ 0.01, analyzed by ANOVA followed by Tukey's post hoc test for multiple comparison.

**Table 5:** Effect of DCM leaf extract of *Gnidia glauca* on navigation distance in HFD-induced obese rats during acquisition training.

Treatment (mg/kgbw)	Navigation distance/path length (cm)			
	Day 1	Day 2	Day 3	Day 4
Normal chow	230.02 ± 20.04^b^	315.14 ± 20.75^b^	269.66 ± 9.08^b^	204.72 ± 9.01^b^
High-fat diet	390.72 ± 20.07^a^	481.10 ± 14.83^a^	448.34 ± 16.90^a^	389.02 ± 13.84^a^
HFD+Orlistat	207.70 ± 23.20^b^	305.90 ± 28.70^b^	257.32 ± 21.17^b^	208.32 ± 21.17^b^
HFD+200 mg/kg	220.30 ± 25.60^b^	299.30 ± 25.40^b^	252.40 ± 23.80^b^	205.70 ± 24.60^b^
HFD+250 mg/kg	218.92 ± 18.61^b^	305.96 ± 21.32^b^	258.30 ± 23.30^b^	193.50 ± 27.70^b^
HFD+300 mg/kg	209.80 ± 16.46^b^	284.90 ± 31.60^b^	233.96 ± 17.51^b^	178.30 ± 23.20^b^

Results are expressed as the means ± SD for five rats per group. The means within the respective columns followed by similar superscript are not significantly different at *p* ≤ 0.01, analyzed by ANOVA followed by Tukey's post hoc test for multiple comparison.

**Table 6:** Effect of DCM leaf extract of *Gnidia glauca* on navigation distance in HFD-induced obese rats during reverse training.

Treatment (mg/kgbw)	Navigation distance/path length (cm)			
	Day 6	Day 7	Day 8	Day 9
Normal chow	253.62 ± 17.44^b^	342.32 ± 8.59^b^	271.60 ± 39.6^b^	235.32 ± 16.85^b^
High-fat diet	431.02 ± 17.19^a^	501.52 ± 15.33^a^	464.64 ± 8.14^a^	447.90 ± 24.70^a^
HFD+Orlistat	249.74 ± 20.12^b^	326.40 ± 16.14^b^	274.78 ± 14.87^b^	230.58 ± 20.87^b^
HFD+200 mg/kg	253.18 ± 21.45^b^	329.92 ± 18.70^b^	284.32 ± 20.11^b^	237.68 ± 17.93^b^
HFD+250 mg/kg	253.10 ± 24.40^b^	333.78 ± 20.16^b^	278.40 ± 24.90^b^	224.70 ± 20.44^b^
HFD+300 mg/kg	232.70 ± 6.90^b^	331.14 ± 6.83^b^	260.92 ± 21.29^b^	215.80 ± 21.22^b^

Results are expressed as the means ± SD for five rats per group. The means within the respective columns followed by similar superscript are not significantly different at *p* ≤ 0.01, analyzed by ANOVA followed by Tukey's post hoc test for multiple comparison.

**Table 7:** Effect of DCM leaf extract of *Gnidia glauca* on swimming speed in HFD-induced obese rats during acquisition training.

Treatment (mg/kgbw)	Swimming speed (cm/s)			
	Day 1	Day 2	Day 3	Day 4
Normal chow	12.22 ± 0.46^a^	11.86 ± 1.07^a^	14.61 ± 0.73^a^	16.77 ± 1.16^d^
High-fat diet	6.21 ± 0.32^b^	7.16 ± 0.35^b^	7.62 ± 0.53^b^	8.05 ± 0.71^e^
HFD+Orlistat	12.47 ± 1.12^a^	11.69 ± 0.88^a^	14.09 ± 1.41^a^	18.26 ± 1.39^cd^
HFD+200 mg/kg	12.73 ± 1.54^a^	12.19 ± 0.56^a^	14.74 ± 1.37^a^	20.31 ± 1.25^bc^
HFD+250 mg/kg	12.57 ± 0.84^a^	12.46 ± 0.42^a^	15.53 ± 1.34^a^	22.24 ± 2.74^ab^
HFD+300 mg/kg	12.13 ± 0.61^a^	12.56 ± 0.24^a^	14.53 ± 1.39^a^	23.67 ± 1.57^a^

Results are expressed as the means ± SD for five rats per group. The means within the respective columns followed by similar superscript are not significantly different at *p* ≤ 0.01, analyzed by ANOVA followed by Tukey's post hoc test for multiple comparison.

**Table 8:** Effect of DCM leaf extract of *Gnidia glauca* on swimming speed in HFD-induced obese rats during reverse training.

Treatment (mg/kgbw)	Swimming speed (cm/s)			
	Day 6	Day 7	Day 8	Day 9
Normal chow	11.12 ± 0.51^a^	11.16 ± 0.12^a^	12.68 ± 0.41^b^	15.12 ± 0.82^d^
High-fat diet	6.54 ± 0.28^b^	6.98 ± 0.35^b^	8.13 ± 1.07^c^	9.02 ± 0.69^e^
HFD+Orlistat	12.03 ± 0.61^a^	11.41 ± 0.46^a^	13.35 ± 0.74^ab^	16.21 ± 1.10^cd^
HFD+200 mg/kg	12.15 ± 1.06^a^	11.59 ± 0.51^a^	13.53 ± 0.64^ab^	17.81 ± 0.40^bc^
HFD+250 mg/kg	11.87 ± 0.71^a^	11.92 ± 0.49^a^	13.86 ± 1.15^ab^	19.93 ± 1.91^ab^
HFD+300 mg/kg	11.22 ± 0.44^a^	11.81 ± 0.30^a^	14.41 ± 0.33^a^	21.26 ± 1.65^a^

Results are expressed as the means ± SD for five rats per group. The means within the respective columns followed by similar superscript are not significantly different at *p* ≤ 0.01, analyzed by ANOVA followed by Tukey's post hoc test for multiple comparison.

**Table 9:** Quantity of phytochemical compounds in DCM leaf extract of *Gnidia glauca*.

RT	Compound name	Relative abundance (%)
21.53	Pyridine-3-carboxamide	10.15 ± 1.58
23.06	Oleic acid	21.05 ± 2.34
24.73	3,5,4′-Trihydroxy-trans-stilbene	13.39 ± 4.06
24.92	Catechins	9.27 ± 2.05
25.44	Octadecanoic acid (stearic acid)	10.73 ± 1.55
25.96	Naringenin chalcone	7.71 ± 1.63
26.35	9,12,15-Octadecatrienoic acid, (Z,Z,Z)-(*α*-linolenic acid)	9.74 ± 2.85
26.98	Luteolin	9.77 ± 2.62
27.90	Eicosapentaenoic acid	7.62 ± 0.89
28.48	Docosahexaenoic acid	7.94 ± 0.44
29.22	Curcumin	16.91 ± 2.30
30.07	Phytol	11.04 ± 1.18
30.24	Quercetin	15.74 ± 1.01
30.79	*γ*-Sitosterol	18.84 ± 1.04
34.92	Cholecalciferol (vitamin D)	9.95 ± 1.42
35.41	Vitamin E	12.25 ± 1.67
35.48	Stigmasterol	7.75 ± 2.23

Summary of compounds identified in *Gnidia glauca* extract with their relative abundance. Results are expressed as the means ± SD for replicate measurement (*n* = 3). RT is the retention time.

## Data Availability

No data was used to support this study.
